# Psychometric Properties of the European Evaluation of Vertigo Scale (EEV) for a Spanish-Speaking Population: A Validation Study

**DOI:** 10.3390/audiolres15040084

**Published:** 2025-07-08

**Authors:** María Alharilla Montilla-Ibáñez, Rafael Lomas-Vega, María del Carmen López-Ruiz, Ángeles Díaz-Fernández, Alfonso Javier Ibáñez-Vera, Ana Belén Peinado-Rubia, Esteban Obrero-Gaitán, Ana Sedeño-Vidal

**Affiliations:** 1 Hospital Universitario de Jaén, Carretera Bailén-Motril s/n, 23007 Jaén, Spain; 2 Departament of Health Sciences, Universidad de Jaén, Campus las Lagunillas, 23071 Jaén, Spain; 3 AFIXA, Calle Baltasar de Alcázar 5, 23008 Jaén, Spain

**Keywords:** vertigo, dizziness, reproducibility of results, validation studies, reproducibility of results

## Abstract

**Background/Objectives**: The objective of this study was to validate the Spanish version of the European Evaluation of Vertigo (EEV) and analyse its test–retest reliability, standard error of measurement (SEM), minimum detectable change (MDC), concurrent validity, and discriminant validity. **Methods**: A cross-sectional validation study was designed. Subjects were recruited from the Otolaryngology Service of the University Hospital of Jaen. Psychometric properties of the EEV were analysed, including the concurrent validity, the SEM, and the MDC. Discriminant validity was calculated using the receiver operating characteristic (ROC) curve. **Results**: The EEV test–retest reliability was nearly perfect (Kappa index = 0.97). The SEM and the MDC were set at 0.56 and 1.10, respectively. Regarding the discriminant validity, the area under the curve (AUC) was 0.831 (95% CI; 0.743–0.899) for the BPPV prediction, the AUC = 0.731 (95% CI; 0.633–0.815) for the disability prediction from the ABC-16 score, and the AUC = 0.846 (95% CI; 0.760–0.911) for the disability prediction from the ABC-6 score. Furthermore, a cut-off point greater than 12 was a good predictor of disability and the fall risk measured with the ABC scale, whereas a value of 11 points was a good predictor for discriminating BPPV patients. **Conclusions**: The Spanish version of the EEV is a valid and reliable instrument for evaluating the clinical symptoms of vestibular syndrome. This instrument demonstrated a nearly perfect test-retest reliability, a low measurement error, and good accuracy in discriminating between patients with vestibular disorders and those with BPPV.

## 1. Introduction

Vertigo patients experience an environmental illusion of movement with respect to their body and vice versa [1]. This alteration is related to a mismatch of the visual, somatosensory, and vestibular systems, which usually includes the appearance of nystagmus and frequently leads to instability and neurovegetative signs, such as nausea [2]. According to Hackenberg et al., 21.6% of the population, or one in five people, occasionally suffer from vertigo, with a peak prevalence ranging from 55 to 64 years of age [3]. In 2021, in the USA, medical expenditures for patients with vertigo totalled USD 48.1 billion [4], whereas indirect costs, such as lost work days, can reach USD 12,542 per patient annually [5]. Recent studies have associated a greater risk of depression and anxiety disorders among vertigo patients [6], which strengthens the idea of considering the impact of vertigo in a wider dimension that must include psychological factors [7,8].

Several instruments have been developed to assess vertigo symptoms and their impact on patients. For instance, Yardley et al. developed the Vertigo Handicap Questionnaire (VHQ), which is a tool for assessing the impact of vertigo on the daily life of patients [9], and the Vertigo Symptom Scale (VSS), which consists of 34 items regarding the frequency and severity of vertigo [10]. Additionally, the Dizziness Handicap Inventory (DHI) was developed by Jacobson et al. as a self-assessment tool designed to quantify the impact of dizziness and vertigo on daily life according to three subscales: functional, emotional, and physical [11]. Other scales, such as the Activities-Specific Balance Confidence (ABC) [12] Scale and the Vestibular Disorders of Daily Living Scale (VADL) [13], were subsequently developed to assess disability secondary to vestibular symptoms, specifically the measurement of the fear of falling in elderly individuals and the impact of balance disorders on independence in routine activities of daily living, respectively.

Another popular tool among clinicians in vertigo assessment is the European Evaluation of Vertigo scale (EEV), developed and validated by Megnibeto et al. [14]. This is a physician-administered questionnaire focused on vertigo symptoms that provides a quantitative score for evaluating vestibular alterations. This tool demonstrated a good interrater reliability (0.93) and satisfactory correlation between the Day-0 scores and the 36-Item Short Form Survey Instrument (SF-36). It consists of five items with equal weight, whose scores range from 0 to 4 on a categorical scale. Administered by a physician, this tool provides a straightforward vertigo assessment, thereby mitigating the risk of bias and misinterpretation often associated with patient self-report questionnaires. This point makes the EEV an important value in comparison with similar tools.

To the best of our knowledge, no Spanish version of the EEV scale has been developed or validated. Considering that the Spanish language is the second most spoken worldwide, the availability of this popular instrument for vertigo assessment is a very valuable development for Spanish healthcare providers in this field. Thus, the aim of this study is to adapt the EEV to the Spanish-speaking population and to assess the psychometric properties of the tool, specifically its test–retest reliability, standard error of measurement (SEM), minimum detectable change (MDC), concurrent validity, and discriminant validity.

## 2. Materials and Methods

### 2.1. Participants

A cross-sectional validation study was designed to meet the objectives of this study. The protocol was approved by the Research Ethics Committee of the Junta de Andalucía, Spain (code SICEIA-2024-001062, date of approval: 30 September 2024). This study was conducted in accordance with the Declaration of Helsinki, good clinical practice guidelines, and all applicable laws and regulations, and written informed consent was obtained from all participating subjects.

**Figure 1:**
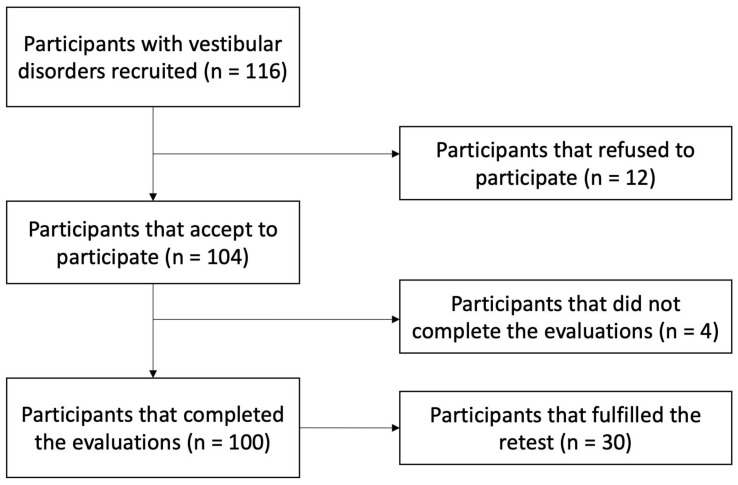
Participant flow diagram.

A sample size calculation was conducted to recruit a minimum of 80 subjects for validity and 20 for test–retest reliability [15]. This study was conducted between May and December 2024. The sample was selected from patients receiving otolaryngology service from the “Complejo Hospitalario de Jaén” (Andalusia, Spain). Recruitment was performed by a physician (Figure 1). Patients over 18 years old, diagnosed with any vestibular disorder (according to Barany Society criteria), whose main complaint was dizziness, and who were able to complete the required questionnaires, met the inclusion criteria. Conversely, participants exhibiting any alteration (such as dementia or Alzheimer’s) that could compromise their understanding of the questionnaires or assessments were excluded. For test–retest reliability, 30 participants were invited to attend a follow-up assessment one week later and were evaluated by the same physician.

### 2.2. Cross-Cultural Adaptation

The recommendations of the IQOLA Project for translating health status questionnaires were used for the translation of the original questionnaire into Spanish. Firstly, two bilingual native speakers translated the original French EEV with the counselling of two experts in the field. Once the translators reached a consensus about the translated version, it was re-translated into French and compared with the original to verify its equivalence concerning clinical, technical, linguistic, and semantic aspects. The final version was pilot-tested with 10 clinicians to detect any difficulties in understanding instructions, items and answers.

### 2.3. Measurements

Once the patients were selected and agreed to participate in this study, we recorded the following demographic data: age, sex, height, weight, and body mass index (BMI).

Considering clinical experience, in addition to its vertigo symptom evaluation properties, the EEV may have properties for measuring balance confidence. To confirm, the Spanish version of the Activities-Specific Balance Confidence Scale (ABC-16) [16] and its short version (ABC-6) were used to assess the balance confidence of the participants. This scale contains 16 items that assess the degree of confidence in balance while performing specific daily tasks. The items are scored from 0% (no confidence) to 100% (complete confidence), with the total score resulting from the average of the items. ABC-16 demonstrated an excellent internal consistency (Cronbach’s *α* = 0.916) and substantial test–retest reliability (ICC = 0.86; 95% CI: 0.74–0.93), with standard error and minimal detectable change values of 8.64 and 16.94, respectively. The short version of the scale (ABC-6) [17] was obtained by extracting Items 5, 6, and 13–16 from the main version, and it showed good psychometric properties.

The Dizziness Handicap Inventory (DHI) was used to assess disability due to dizziness and was culturally adapted to the Spanish population [18]. The DHI contains 25 items with three response options, “yes”, “no”, or “sometimes”, which are quantified with 4, 0, or 2 points, respectively. The DHI provides three scores: functional (nine items), physical (seven items), and emotional (nine items). The scores of the different dimensions studied are categorized as “no disability” if the functional and emotional dimensions score below 15 points and the physical dimensions score below 10 points, “moderate disability” if the functional and emotional dimensions score between 15 and 24 points and the physical dimension scores between 10 and 16 points, and “severe disability” if the functional and emotional dimensions score between 24 and 36 points and the physical dimension scores between 17 and 28 points. An overall total score is also considered, with “mild disability” ranging from 0 to 30, “moderate disability” ranging from 31 to 60, and “severe disability” ranging from 61 to 100 [18]. The DHI total score results in four severity grades ranging from 0 to 3; scores ranging from 0 to 14, 16 to 34, 36 to 52, and 54 or greater indicate “none” and a “mild”, “moderate”, and “severe” handicap, respectively. According to Jacobsen et al. [11], a minimum change of 18 points is considered a true change in the DHI score.

### 2.4. Statistical Analysis

A descriptive analysis was performed by calculating the means and standard deviations for the continuous variables and the frequencies and percentages for the categorical variables. The Kolmogorov–Smirnov test was used to verify the normal distribution of the continuous variables, and the Levene test was used to test the homoscedasticity in the samples. The confidence level was set at 95% (*p* < 0.05).

For the reliability and discriminant validity analysis, Version 23.1.7 of MedCalc^®^ Statistical Software was used (MedCalc Software Ltd., Ostend, Belgium; https://www.medcalc.org; 2025). For the reliability, a Kappa coefficient weighted by quadratic weights was used [19]. Weighted Kappa is asymptotically equivalent to an intraclass correlation coefficient [20]. Kappa coefficient values of <0.00, 0.00–0.20, 0.21–0.40, 0.41–0.60, 0.61–0.80, and 0.81–1.00 indicated null, insignificant, discreet, moderate, substantial, or almost perfect reliability, respectively [21].

The standard error of measurement (SEM) was calculated as the baseline standard deviation (SD) (σbase) minus the square root of (2-Rxx), where Rxx is the reliability index (Kappa). The minimum detectable change (MDC) was calculated from the SEM formula as MDC95 = 1.96 ∗ σbase ∗ $\sqrt{\left(2-Kappa\right)}$, where 1.96 is the z value corresponding to the 95% confidence interval (MDC95). The MDC provides a good tool for translating the reliability index into units of change in the instrument.

For the analysis of the concurrent validity, Version 27.0 of the Statistical Package for Social Sciences (SPSS Inc., Chicago, IL, USA) was used. For this analysis, the Spearman’s Rho correlation coefficients between the total EEV score and the DHI and ABC subscales were calculated. Following Cohen’s criteria, correlations lower than 0.30 were considered poor, those between 0.30 and 0.50 were considered moderate, and those greater than 0.50 were considered strong [22].

The capacity of the EEV to discriminate between patients with and without a disability was calculated using receiver operating characteristic (ROC) curves. The subject classification of patients with a disability was conducted based on the cut-off point of the scores obtained in ABC-16 [23] and ABC-6 [17] to predict disability, and the total score obtained in the EEV was evaluated as a variable. The same procedure was conducted to discriminate between subjects with BPPV and those with other pathologies. In the ROC curve, the fraction of true positives (sensitivity) was represented as a function of the fraction of false positives for different cut-off points. The area under the curve (AUC) was also calculated as a measure of the ability of the score to discriminate between the two diagnostic groups (patients with or without risk of fall and those with or without BPPV). The AUC was considered statistically significant when the 95% confidence interval did not include 0.5 [24]. Values between 0.5 and 0.7 indicated low accuracy, those between 0.7 and 0.9 indicated good accuracy, and those greater than 0.9 indicated high accuracy [25].

## 3. Results

Among the 100 patients, 54 had Ménière’s disease, 16 had vestibular migraine, and 30 had BPPV. The data of the sample of the participants are shown in Table 1.

### 3.1. Test–Retest Reliability

The total score had a Kappa index of 0.97 (95% CI; 0.91–1.00), which could be considered nearly perfect agreement. Based on this index, the SEM was 0.56, and the MDC was 1.10. These findings indicate that when the total score varies between 0 and 20 points, the error can be less than 1 point, and the minimal change is 1 point.

### 3.2. Concurrent Validity

The correlation between the total score of the EEV and other measurements of dizziness or the balance confidence (Table 2) was strong when compared with the balance confidence measurements, moderate when compared with the DHI-Emotional measurements, and poor when compared with the other DHI subscales or the DHI total score.

### 3.3. Discriminant Validity

**Figure 2:**
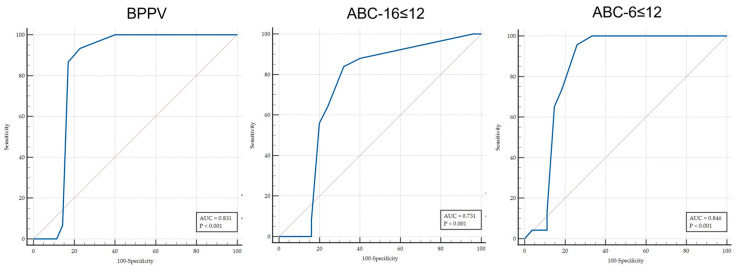
ROC curves.

The ROC curve analysis revealed an AUC of 0.831 (95% CI: 0.743–0.899) for the BPPV prediction, an AUC of 0.731 (95% CI: 0.633–0.815) for the disability prediction from the ABC-16 score, and an AUC of 0.846 (95% CI: 0.760–0.911) for the disability prediction from the ABC-6 score, all of which demonstrated good accuracy (Figure 2). A cut-off score of >12 on the EEV was a good predictor of the disability and fall risk measured with the ABC, and a score of 11 points was a good predictor for discriminating the BPPV patients. The predictive values are shown in Table 3.

## 4. Discussion

The EEV is a popular tool among physicians for the assessment of vertigo symptoms, although no Spanish version has yet been validated. The Spanish version of this tool (Supplementary Materials) demonstrated a nearly perfect test–retest reliability, a strong correlation with other balanced confidence measurements, and a high accuracy in predicting disability and the risk of falls due to vertigo. It also showed a good predictive capacity for discriminating BPPV patients, which is noteworthy for clinicians.

The original validation method by Megnigbeto et al. utilized SF-12 for the generic quality of life, the vertigo evaluation scale of the Direction de la Pharmacie et du Medicament (DPHM), and the American Academy of Otolaryngology-Head and Neck Surgery scale (AAO) as a comparison to determine the construct, convergent, and discriminant validity [14]. However, the present study compared ABC-16, ABC-6, DHI, and VHQ, which are the more specific and developed scales for vertigo. Concurrent validity was assessed in the original version via the Pearson correlation, which revealed a statistically significant but moderate correlation (r > 0.40) with the “mean number of attacks” of the DPHM scale. However, the remaining correlations, such as those with the AAO and its sub-scores, were weak. Otherwise, the Spanish version demonstrated a strong correlation with the balance confidence measurements and a moderate or poor correlation with the DHI and its sub-scores, which means that the EEV is better at detecting balance confidence than at measuring disability due to vertigo. The method used by the original authors for the reliability assessment calculated the mean differences from the baseline and after seven days for some EEV sub-scores, determining that the changes were “not significant” (*p* value < 0.20). The Spanish version demonstrated an almost perfect reliability (Weighted Kappa = 0.97), demonstrating a better value for this property. Another interesting point of the original version is the interrater reproducibility, which was stated as excellent. New psychometric properties were assessed as a new contribution in the Spanish version: MDC and SEM were established, and the discriminant validity was calculated via an ROC curve analysis, which revealed a high accuracy for BPPV prediction and disability prediction.

A previous cross-cultural adaptation of the EEV was developed in the Turkish language [26]. This version demonstrated several similarities with the Spanish version: excellent test–retest reliability (ICC = 0.835; IC 0.72–0.90), which is slightly lower than that of the Spanish version, with an SEM of 1.34 for the total score (SEM = 0.56 for the Spanish version) and an MDC of 3.71 (MDC = 1.1 in the Spanish version). The Turkish version also showed an acceptable construct validity and a moderate correlation with other scales, such as the Turkish version of the Vertigo Symptom Scale [26].

The EEV has been used in several experimental studies for the assessment of vertigo symptoms. Eryaman et al. investigated the effectiveness of hypertensive treatment in vertigo patients, using the EEV and the VHQ scales to assess vertigo symptoms [27]. In that study, a statistically significant difference (*p*-value = 0.001) was obtained between the experimental and control groups (four points between the means of the groups). According to our data, the results of Eryaman et al. appear to be relevant, as our SEM was lower than the differences between the groups, both at the baseline and after treatment, and the MDC was also lower than the differences obtained. This indicates that the possible errors in the measurement of the EEV and the ability of the scale to detect changes in this study did not affect the validity of the results obtained, indicating that the hypertensive treatment was effective.

Another study investigated the effects of COVID-19 infection on hearing and the vestibular system, using the EEV for the assessment [28]. The mean score of the EEV in the group that was positive for COVID-19 was 4.5, indicating a statistically significant difference with the control group (*p*-value < 0.05). Considering that this value was higher than the MDC of both versions (Turkish and Spanish), a relevant difference in the severity of the vertigo symptoms between the groups can be considered. Nonetheless, Buendía-Pajares et al. reviewed the scales mostly used to evaluate vertigo patients prior to vestibular rehabilitation [29]. Among the several instruments, the EEV and VSS were recommended for measuring vertigo symptoms and their intensity. The authors emphasized the importance of using the EEV scale to measure symptoms and their severity in patients with instability or dizziness during the week leading to the evaluation [29]. In summary, the EEV is a useful tool for the assessment of vertigo symptoms and is recommended by physicians for this purpose.

This study has several limitations. First, the results of our study are based on a cohort from a single hospital that is a national reference in Ménière’s disease, which requires caution when establishing a generalization of the results. Second, the major limitation of our study is that a limited number of pathologies were included, and we did not differentiate between the types of pathologies. Studies verifying the instrument’s validity under other vestibular conditions would also be desirable. Finally, although our analysis considered some psychometric properties that were not analysed in the original EEV, other properties that were not analysed should be considered in future studies.

## 5. Conclusions

In conclusion, the EEV demonstrated good test–retest reliability, high precision, and low measurement error. With respect to discriminant validity, the EEV demonstrated good accuracy in predicting the disability, as measured by the ABC-16 and ABC-6 cut-off points, and in discriminating the BPPV patients. The EEV has good overall psychometric properties and is suitable for assessing signs such as the illusion and duration of movement, motion, intolerance, neurovegetative signs, and instability in patients with vestibular disorders.

## Figures and Tables

**Table 1:** Basic characteristics of the sample.

		Count	Mean	Standard Deviation
Diagnostic	Ménière’s disease	54		
	Vestibular migraine	16		
	BPPV	30		
Gender	Male	58		
	Female	42		
Age (years)			57	8
Weight (kilograms)			70.5	9.4
Height (metres)			1.71	0.06
Body mass index			23.94	2.09
DHI_Total_Score			45.56	5.83
ABC_16			63.29	14.98
EEV_Score			11.74	2.16

**Table 2:** Concurrent validity between the EEV score and the rest of the variables.

**Variable**	**Spearman’s Rho Coefficient**	**Correlation**	***p*** **-Value**
DHI_Emotional	−0.422	Moderate	<0.001 ***
DHI_Physical	0.257	Poor	0.010 *
DHI_Functional	−0.284	Poor	0.004 **
DHI_Total	−0.179	Poor	0.075
ABC_16 items	−0.538	Strong	<0.001 ***
ABC_6 items	−0.501	Strong	<0.001 ***

ABC: Activities-Specific Balance Confidence Scale; DHI: Dizziness Handicap Inventory; * *p* < 0.05; ** *p* < 0.01; *** *p* < 0.001.

**Table 3:** Predictive values for different cut-off points of the European Evaluation of Vertigo (EEV) Scale to predict disability measured with the ABC scale and to discriminate between the BPPV and other patients.

EEV Cut-Off	Sen	95% CI	Spe	95% CI	+LR	95% CI	−LR	95% CI	+PV	95% CI	−PV	95% CI	Criterion
≤12	84.00	70.9–92.8	68.00	53.3–80.5	2.63	1.72–4.00	0.24	0.12–0.46	72.4	63.3–80.0	81.0	68.7–89.2	ABC-16 ≤ 67
≤12	95.65	85.2–99.5	74.07	60.3–85.0	3.69	2.34–5.82	0.059	0.015–0.23	75.9	66.6–83.2	95.2	83.6–98.7	ABC-6 ≤ 55
≤11	93.33	77.9–99.2	77.14	65.6–86.3	4.08	2.63–6.35	0.086	0.023–0.33	63.6	53.0–73.1	96.4	87.6–99.0	D = BPPV

Sen: sensitivity; Spe: Specificity; LR: Likelihood Ratio; PV: predictive value; D: diagnostic; BPPV: Benign Paroxysmal Postural Vertigo.

## Data Availability

The data presented in this study are available on request from the corresponding author because of privacy concerns.

## Supplementary Materials

The following supporting information can be downloaded at https://www.mdpi.com/article/10.3390/audiolres15040084/s1, Spanish Version of the European Evaluation of Vertigo (EEV).
