# Supplementary material for: Psychometric Properties of the European Evaluation of Vertigo Scale (EEV) for a Spanish-Speaking Population: A Validation Study
Source: Audiol Res. 2025 Jul 8;15(4):84. doi: 10.3390/audiolres15040084 (PMC12286084; doi:10.3390/audiolres15040084)
Supplement: Supplementary file 1 [file audiolres-15-00084-s001.zip › audiolres-3686526-supplementary.pdf]

Hospital Universitario de Jaén

Universidad de Jaén

EVALUACIÓN EUROPEA DEL VÉRTIGO (EEV)

1. ILUSIÓN DE MOVIMIENTO
    0. No existe ilusión
    1. ………………………………………………….
    2. Sensación de balanceo hacia la derecha o hacia la izquierda, hacia arriba o hacia abajo, pequeños dolores de cabeza,
    3. …………………………………..
    4. Sensación de giro de uno mismo o del entorno

2. DURACIÓN DE LA ILUSIÓN DE MOVIMIENTO
    0. Nada
    1. Menos de 1 minuto
    2. De 1 minuto a 1 hora
    3. De 1 hora a 3 horas
    4. De 3 horas a 24 horas

3. INTOLERACIA AL MOVIMIENTO
    0. Sin intolerancia de movimiento
    1. Raramente o unas pocas
    2. A veces o moderadamente
    3. A menudo
    4. Siempre o intensamente

4. SIGNOS VEGETATIVOS
    0. No hay signos vegetativos
    1. La sensación de náusea no corresponde con el ataque de vértigo
    2. La sensación de náusea se asocia al ataque de vértigo
    3. La sensación de náusea se asocia con uno o dos episodios de vértigo
    4. Vómito intratable

5. INESTABILIDAD
    0. No existe inestabilidad
    1. Inestabilidad sin caídas no limitación de las actividades de la vida diaria
    2. Inestabilidad sin caídas pero con limitación de las actividades de la vida diaria
    3. Inestabilidad con caídas ocasionales estando de pie o andando
    4. Inestabilidad y caídas con solo ponerme de pie

Montilla-Ibáñez MA, Lomas-Vega R, Ibáñez-Vera AJ, Peinado-Rubia AB, Obrero-Gaitán E, Sedeño-Vidal, A. Psychometric properties of the European Evaluation Vertigo Scale (EEV) for the Spanish-speaking population: A validation study. 2025.
